# Generative design of 3D printed hands-free door handles for reduction of contagion risk in public buildings

**DOI:** 10.1007/s12008-021-00825-6

**Published:** 2022-01-12

**Authors:** F. De Crescenzio, M. Fantini, E. Asllani

**Affiliations:** 1 Department of Industrial Engineering, University of Bologna, 47121 Forlì, Italy; 2 Romagna Tech s.c.p.a., 47121 Forlì, Italy; 3 University of Bologna, 47121 Forlì, Italy

**Keywords:** Covid 19, Reverse Engineering, Generative Design, Additive Manufacturing

## Abstract

During the emergency caused by COVID 19 evidence has been provided about the risk of easily getting the virus by touching contaminated surfaces and then by touching eyes, mouth, or nose with infected hands. In view of the restarting of daily activities in presence, it is paramount to put in place any strategy that, in addition to social distancing, is capable to positively impact on the safety levels in public buildings by reducing such risk. The main aim of this paper is to conceive a design methodology, based on a digital, flawless, and sustainable procedure, for producing human-building interfacing solutions that allow anybody to interact in a safer and more comfortable way. Such solutions are focused on the adaptation of existing buildings features and are thought to be an alternative to sensor based touchless technology when this is not applicable due to economic or time constraints. The process is based on the integration of digital technologies such as 3D Scanning, Generative Design and Additive Manufacturing and is optimised to be intuitive and to be adaptive, hence, to be replicable on different kinds of surfaces. The design concept is finalised to generate automatically different products that meet geometry fitting requirements and therefore adapt to the specific geometries of existing handles. A specific case on Hands Free Door Handles is presented and the results of manufacturing and preliminary validation process are provided and discussed.

## Introduction

Acute Respiratory Syndrome Coronavirus 2 (SARS-CoV-2) is the new Corona Virus identified for the first time on humans at the end of 2019. COVID-19 is the name given to the disease associated with the virus. Such microorganism stepped in our lives and spread so quickly that the health system was not ready to cope with it. Therefore, in the last year Governments of all over the world started to implement measures to reduce the spreading of contagion. In this context, one of the main issues has been the scarce knowledge about the modes of transmission. Thus, in the first months of the pandemic, several studies have been conducted in order to gather a clearer understanding of this process. One of the first studies published in literature right after the first identification of the new Corona Virus has demonstrated that its diffusion is facilitated by personal contact [1]. Hence, the idea that droplets, contaminated hands, or surfaces are the main means of transmission started to be a certainty. Van Doremalen et al., in 2020, through lab experiments, proved that the median half-life of virus on plastic and still is, respectively, 6.8 and 5.6 h. Anyway, they conclude that the virus can remain viable and infectious in aerosols for hours and on specific surfaces even up to days [2]. Other studies scientifically confirm that the virus can survive on surfaces for several hours [3, 4]. In a study conducted in a hospital in Italy, where air and surfaces contamination have been observed, it emerged that the handles are among the most contaminated contact surfaces, since the virus has been identified in 25% of the total handles observed [5].

However, contamination of surfaces was a problem explored even before the advent of Corona Virus. In a study conducted two busy hospitals in UK, Wojgani et al. (2012) concluded that the design of the door handles is one of the factors influencing the bacteria contamination level. In detail, they report that lever handles display the highest level of contamination, compared to other surfaces and other handle typologies in the sample [6]. Not surprisingly, in a study on the most frequently touched surfaces in a COVID-19 hospital ward, the bathroom door handles have been recognized as alert factors of high contamination risk [7].

If not correctly sanitised, as recommended by OMS (OMS 200), contaminated hands are a means of transmission, especially for those who frequently touch their faces. As demonstrated in a behavioural study involving university students in medicine in South Wales, such frequency is considerable. In mean, each of the 26 participants to the experiment has been observed to touch his/her face 23 times per hour. In detail, 44% of the total touches has regarded the mucous membrane (36% the mouth, 31% the nose and 27% the eyes) [8].

To sum up, there is significant evidence that COVID is highly contagious through infected surfaces, and, among these surfaces, the door handles in public buildings represent one of the most touched and contaminated surfaces. Currently, the strategy to reduce the risk of contagion by contact surfaces is based on the use of hand sanitisers, such as alcoholic sanitising solutions, or lattice gloves. Such solutions present different limits. Firstly, in a prolonged use they can damage the skin. Secondly, they rely on the user and on the frequency he/she sanitises his/her hands. Moreover, these are non-sustainable solutions, given the production of plastic for disposable gloves or gel bottles.

This paper aims to develop design solutions for door handles to reduce the indirect risk of contagion due to surface contact and to provide a semi-automatic design methodology based on digital technologies, such as Reverse Engineering, Generative Design and Additive Manufacturing for a fast, sustainable, cost effective and delocalised reconversion of public building in safer environments. The main idea consists in transferring the grab, push, and pull functions from the hand to other parts of the body that are usually covered by clothes and that are not usually in contact to our faces, such as the forearm. In the recent past, similar solutions have been introduced in new building constructions. For example, in the faucets of public restrooms. Otherwise, in the most technologically advances, touchless solutions based on photocells and electric actuation of doors or faucets, or other devices are implemented. Nevertheless, such hands free or touchless interaction devices can be found only in the most recent buildings and when this is economically sustainable, for example in the airports. Now, all the public buildings, such as schools, universities, gyms, public transportation, public offices, and any other high-risk workplace need to be quickly adapted. Some similar solutions have been provided successfully during the first period of the pandemic but are based on fixed design solutions that can be adapted only to a set of specific existing geometries [9]. In this paper, a methodology to generate even a single printable CAD model, but automatically and with a high degree of customization is proposed.

In the next sections, the process (Sect. 2—Methodology) is firstly described. Then the implementation of the process on a demonstration case consisting in a lever handle scanned on a door inside the building where our university classes and professor offices are located is presented (Sect. 3—The Lever handle Case). Finally, the results and conclusions are discussed.

## Methodology

**Fig. 1:**
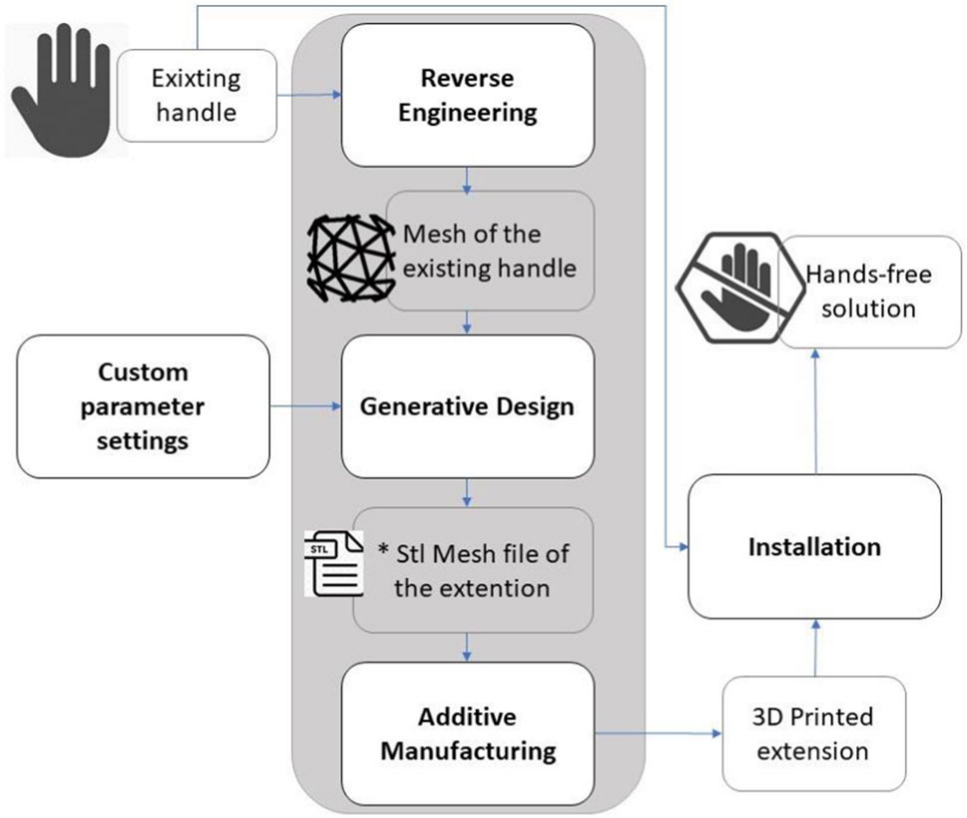
The hands-free solution generation procedure

The solution proposed in this paper is based on the idea to design and manufacture an extension for handles that, once correctly installed, allows the user to open or close the door by using the elbow of forearm instead of the hand. Therefore, an analysis of the door handle design space has been performed, and it has been observed that the main designs of handles that need direct contact with hands and that are used in private and public buildings are the lever handles, the pull handles, and the knob handles. Hence, a procedure that can be initiated once a specific handle is scanned and that automatically delivers different families of solutions for different handle design concepts (lever, knob, pull) has been conceived. The entire design and manufacture methodology is depicted in Fig. 1. Such approach has already been adopted in other domains, such as the medical domain, where the realization of products perfectly fitting a custom free form shape is a main requirement. In medicine the free form surface is the patient anatomy, and the product can be a customised orthosis or prosthesis. For example, a similar approach is adopted in [10] for the automatic generation of 3D printable foot orthoses starting from the scan of the foot plant of the patient. In [11] the authors describe the process for the generation of a protective mask adapted to the scan of the customer’s face. Specifically, they point out the use of off-the-shelf devices and the possibility to have a practitioner-friendly approach. In [12, 13] more applications for foot orthoses are described.

The methodology here described aims at a complete automatic process. It is composed of three main functional blocks representing three different and sequential phases. Firstly, the Reverse Engineering block represents the digitalization of the existing handle. It can be performed with different kinds of devices. Among the different solutions proposed for low cost, portable off-the-shelf scanning system, the Sense 3D Scanner has been selected. The Generative Design block is the core of the process. The main idea is to automate as much as possible the 3D modelling phase based on a semi-automatic CAD generation algorithm that provides the ready-to-print geometric model of the extension once the scan is available. This allows generating not a single design solution, but a library of solutions by conceiving a generative design algorithm for each design of handle and by adapting such algorithm to the specific dimension, section geometry, curvature, or any other specific features of the single handle. The design and 3D modelling process isare optimised for the 3D print of the extension, which represents the third block of the process. Additive Manufacturing has demonstrated to be a valuable technology to face the emergency due to COVID 19 when the entire world has experienced a peak in the demand of PPE (Personal Protection Equipment), as well as medical devices such as valves for ventilators [14]. To address this issue, makers all over the world have exploited the possibility to quickly produce additional components without setting up new production lines and everywhere in the world a 3D printer is located. Also, the hands-free extension generation process proposed in this work is intended to be a completely customisable solution that can be implemented wherever a low-cost scanner and a 3D printer are available without the need of creating specific manufacturing lines, even in low scales of production, and with a limited investment compared to other solutions.

**Fig. 2:**
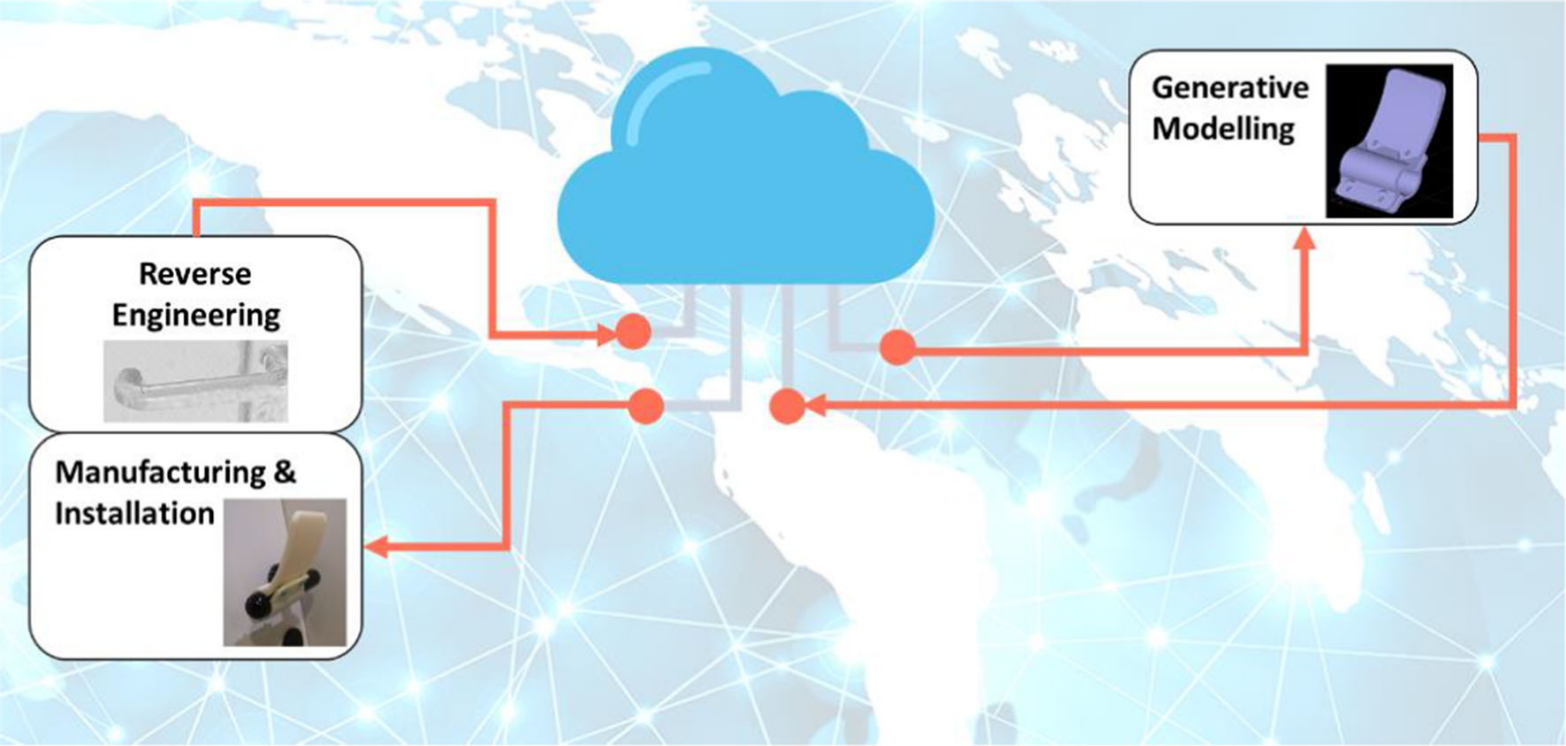
Delocalization of geometric modelling function through generative design

One of the advantages of using generative design for the hands-free solution generation is the possibility to delocalise the modelling function from the scanning, manufacturing, and installation functions. While the latter need to be performed at the building site, the modelling function is intended to be automatic as a service that can be provided at worldwide level in a connected environment for a customised product (Fig. 2).

Having observed the high occurrence of lever handles, this type of handle has been selected for the demonstrative solution. Hence, the lever handle is the single case that will be used to develop the automatic generation tool that is the most diffused type of handle.

## The lever handle demonstrator

The hands-free solution generation procedure has been developed in detail and tested for a demonstrator regarding a specific type of handle: the lever handle. This is the most common one and is produced with different geometries sharing the same architecture. Geometries mainly differ in the section profile, that can be circular, oval, polygonal or free form, in the length of the lever, in the presence of the hook or in the tapering. To deliver a robust and adaptive solution, a generative design algorithm has been conceived and implemented. Once the mesh of the scanned handle is uploaded, in a few minutes the algorithm generates the *stl file of the extension for the lever handle according to the specific geometry. In the next subsections, the three steps needed to deliver the solution: 3D scanning (Reverse Engineering), 3D model generation (Generative Design) and 3D printing (Additive Manufacturing) have been described in detail.

### 3D scanning of the handle

**Fig. 3:**
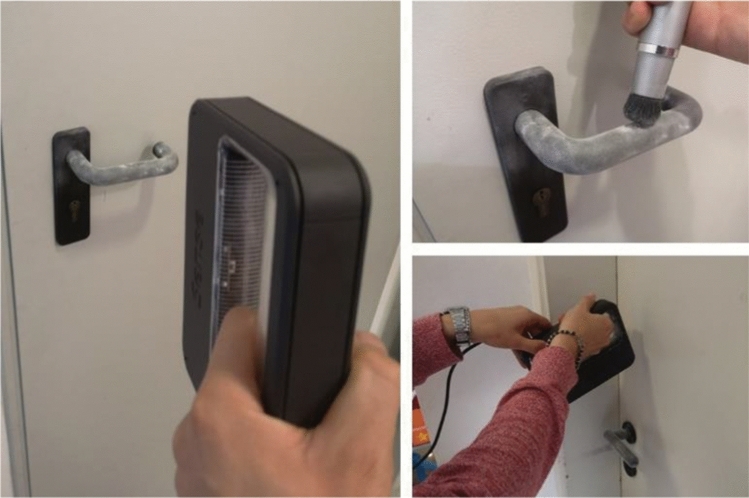
3D Scanning phase

The hardware for scanning the handle used in this work is the Sense 3D Scanner provided by Cubify. This scanner projects an invisible infrared laser onto the object and senses the depth and the colour through two cameras, one infrared and one RGB camera. It works well for objects of limited size such as door handles. It is a handheld device that the user must slowly move while pointing at the object to scan in order to progressively acquire the points on its surface. For scanning the handle, its surface has been covered with matte white powder and the scanner has been positioned at around 40 cm (Fig. 3). As usual in optical scanner, it is not always possible to view the surface from all sides since parts of it cannot be framed due to the presence of other objects that cover it. In the case of handles, for example, the surface of the door is very close to the rear part of the handle. To solve this issue, the acquisition includes also the views from the top and from the bottom and a *close holes* procedure is then implemented during the post-processing of the mesh. No major issues or difficulties have been encountered for this geometry and the use of the automatic functions in the support software, such as *solidify*, allowed to gather a mesh perfectly usable in the modelling process.

### 3D Model generation

**Fig. 4:**
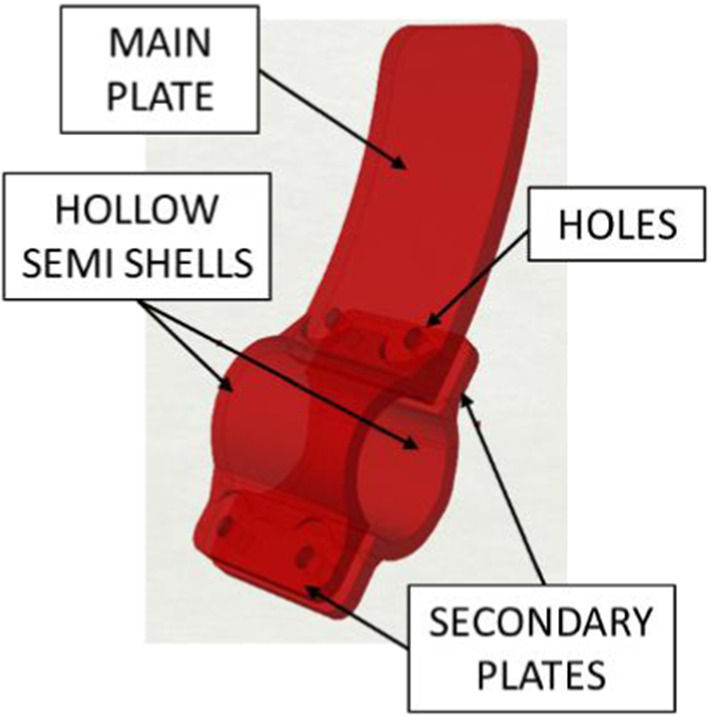
Conceptual lay out of the handle extension

Generative Design allows creating 3D models through algorithms defined by the user with the aim to generate different geometries by varying quantitative or qualitative parameters. Compared to classic CAD modelling processes, the Generative Design procedure allows to store the design intent in the algorithm and to always generate new shapes without the need of building a new model from the scratch. In product and architectural design, this approach is popular for creating new and out of the scheme shapes extending the creativity of designers [15]. To implement this shape generation approach in CAD platforms, designers can define the algorithms by coding in a Programming Language and using the scripts for the CAD functions. Otherwise, designers can use Visual Programming Languages (VPL) that consist of iconic functions, which can be manipulated on a canvas offering a faster learning alternative to scripting [16, 17]. One of the most popular VPL for Generative Design of CAD models is Grasshopper, a plug-in of Rhinoceros 3D. Therefore, the CAD of the handle extension in this paper is generated through a series of subroutines implemented in subcircuits designed in Grasshopper. The conceptual layout of the extension for lever handle is inspired to solutions already proposed by leader 3D printing system manufacturers [18]. As depicted in Fig. 4 it includes two main functions, which are the interface with the existing surface and the mechanism to transfer the handle turning/pushing/pulling functions to the forearm. The first function is performed through a hollow shell in which the cavity embeds the handle section. To be assembled the shell needs to be manufactured into two semi shells that have to be fixed through integrated plates allocating the screws. To interact with the extension the user needs a sufficiently large flat surface (main plate) and a manoeuvring space allowing him to insert the forearm between the handle and the door, to push it and therefore provoking the rotation of the existing handle and eventually pull the door towards himself.

**Fig. 5:**
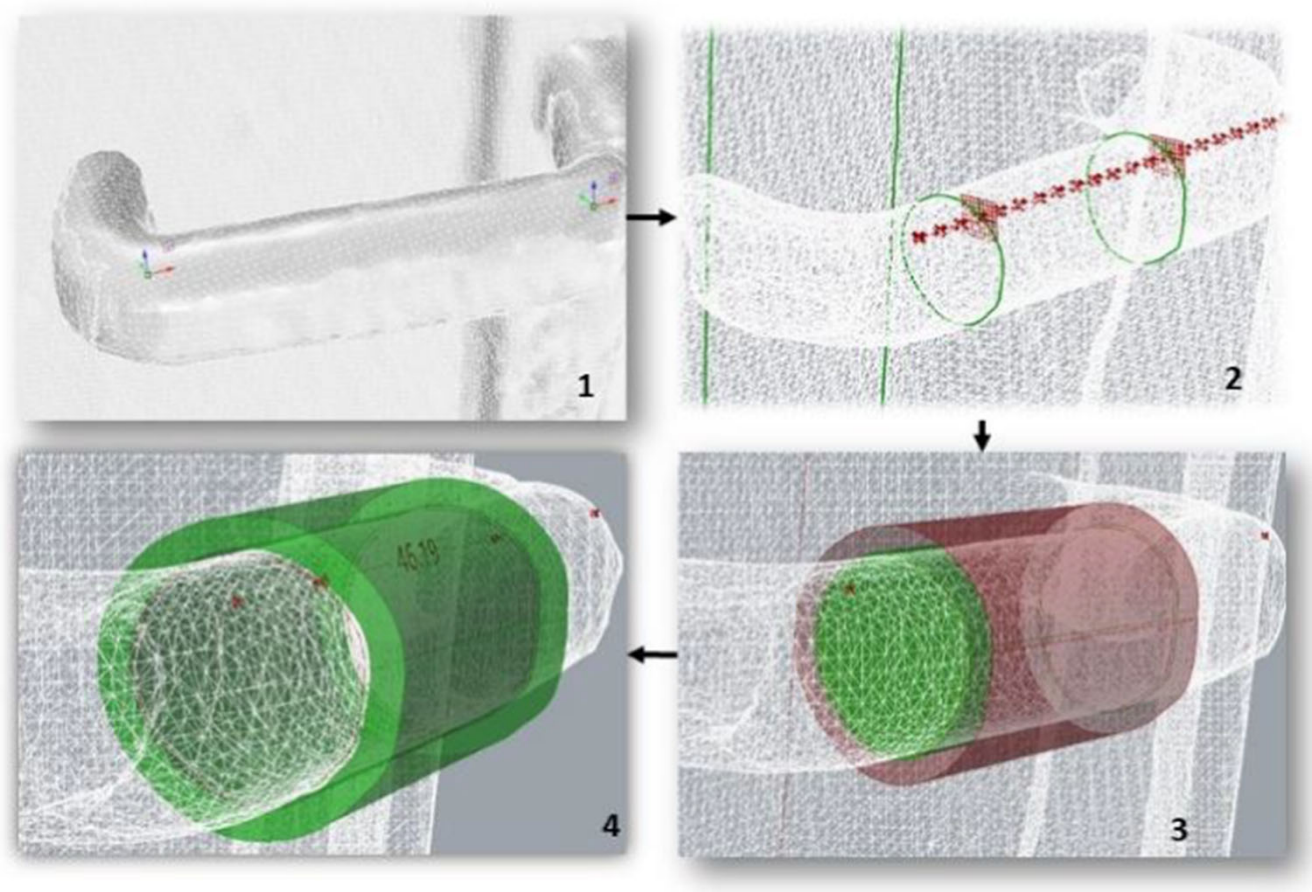
Generation of the tubular hollow shell: (1) Handle scan and two initial points, (2) Contour sections and intermediate sections positions, (3) Offset of the handle surface, (4) Tubular solid with appropriate backlash with the mesh

**Fig. 6:**
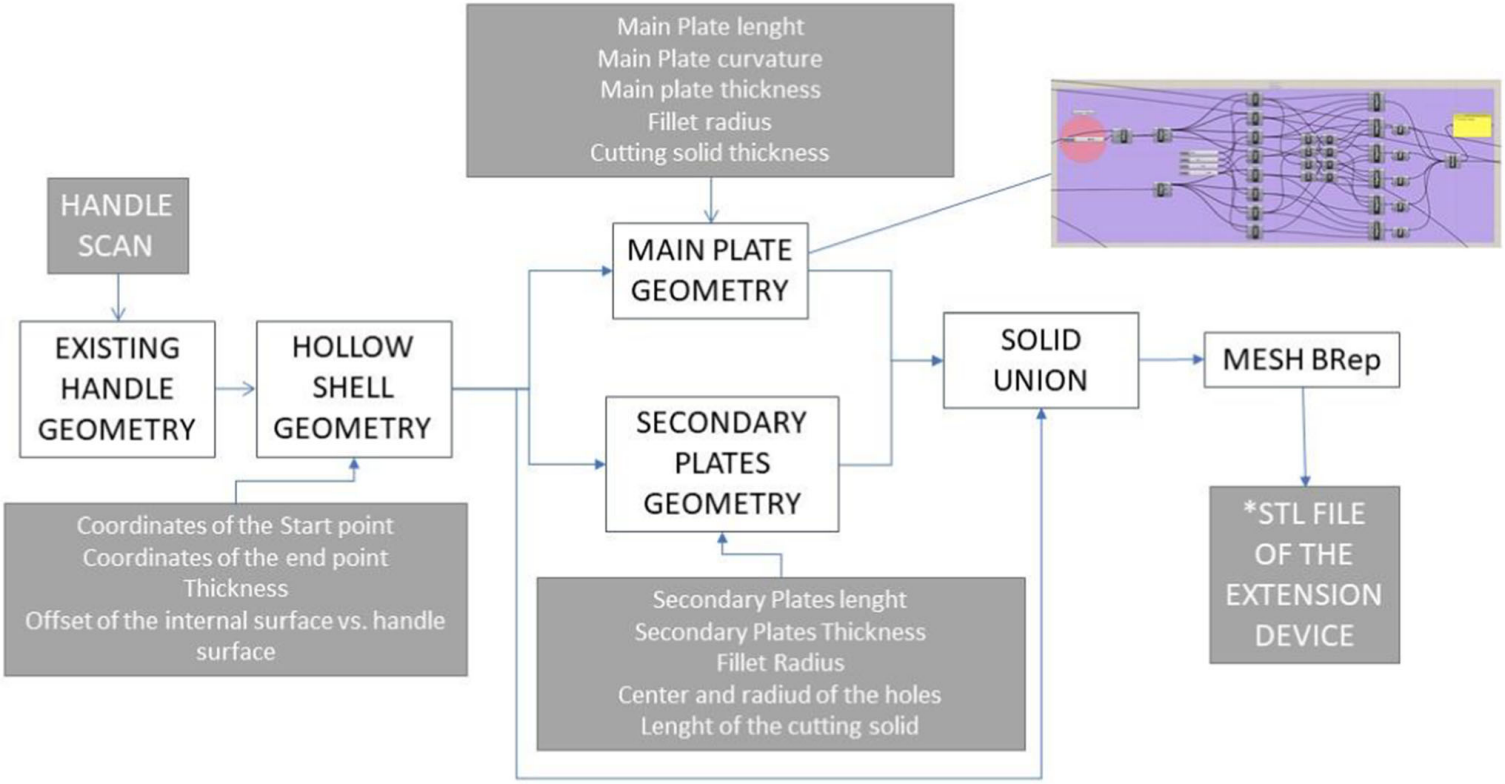
Workflow for the Generative Design of the lever handle hands-free extension

Since the entire graph in Grassopper can be divided into a number of subcircuits, each of them generating a specific part of the model, different circuits have been conceived to model the shells and the plate components. The first circuit, that generates the main body of the shells, starts with the mesh of the existing handle and two points selected on the mesh defining the location of the two opposite sections (start and end point) of the hollow part (Fig. 5). As depicted in Fig. 6, further subcircuits generate the main plate and the secondary plates for the allocation of the screws.

The generation process is made interactive through the definition of a set of dimensioning parameters (for example the thickness of the plates) that are variables in a certain domain. Such parameters are connected to *number sliders* whose values can be changed interactively and intuitively. This approach also helps keep the project adaptable, for example, to different 3D printing materials that can require specific thickness.

**Fig. 7:**
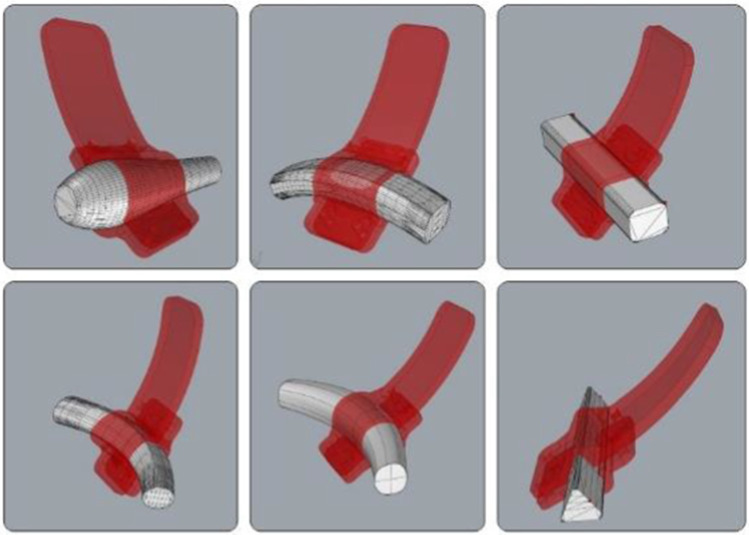
Results of tests on different lever geometries

As a result, an infinite library of adaptable devices completely independent from the shape and curvature of the lever handle is virtually available. Some examples of extensions automatically generated for different possible lever geometries are shown in Fig. 7. Starting from a set of geometries with different morphologies (round, square, triangular or polygonal), curvatures or not constant sections, the printable 3D model of the auxiliary extension is generated in few seconds.

### 3D Printing

Four specimens of lever handle extensions have been 3D printed by means of a Fused Deposition Modelling (FDM) system. The Additive Manufacturing system adopted is the Fortus 250 mc provided by Stratasys and the material used is the ABSplus (tensile Strength 34 MPa). The Fortus 250 deposits in each layer, together with the build material, a soluble support that can be simply removed in a post-processing phase, making this technology suitable for printing complex shapes.

**Fig. 8:**
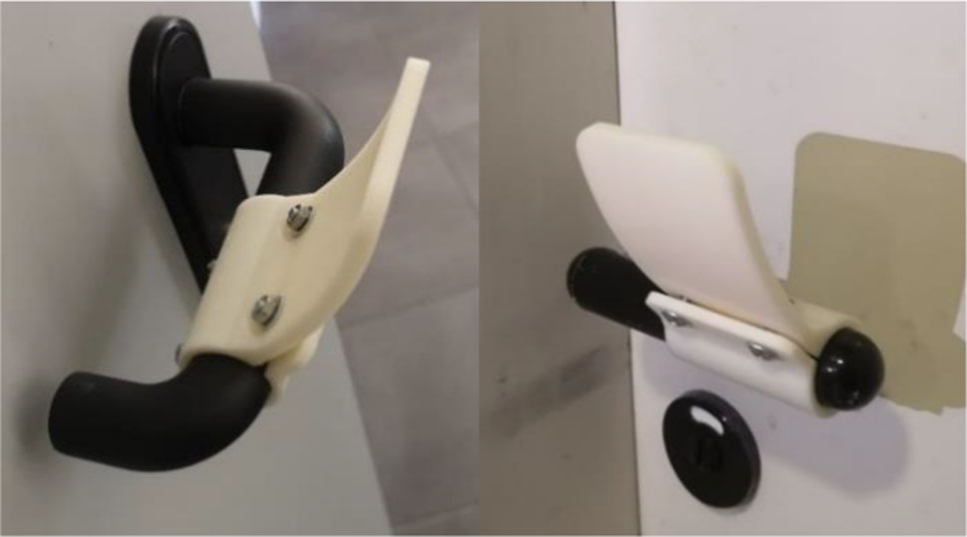
Lever handle with extension: Lever Type 1 (left), Lever Type 2 (right)

Hence, two lever handles, lever type 1 (with hook) and lever type 2 (less rough surface), with different section dimensions and surface roughness have been scanned (Fig. 8).

In the pre-processing phase, among the different printing parameters, the building orientation and of different filling options have been varied to obtain different options.

**Fig. 9:**
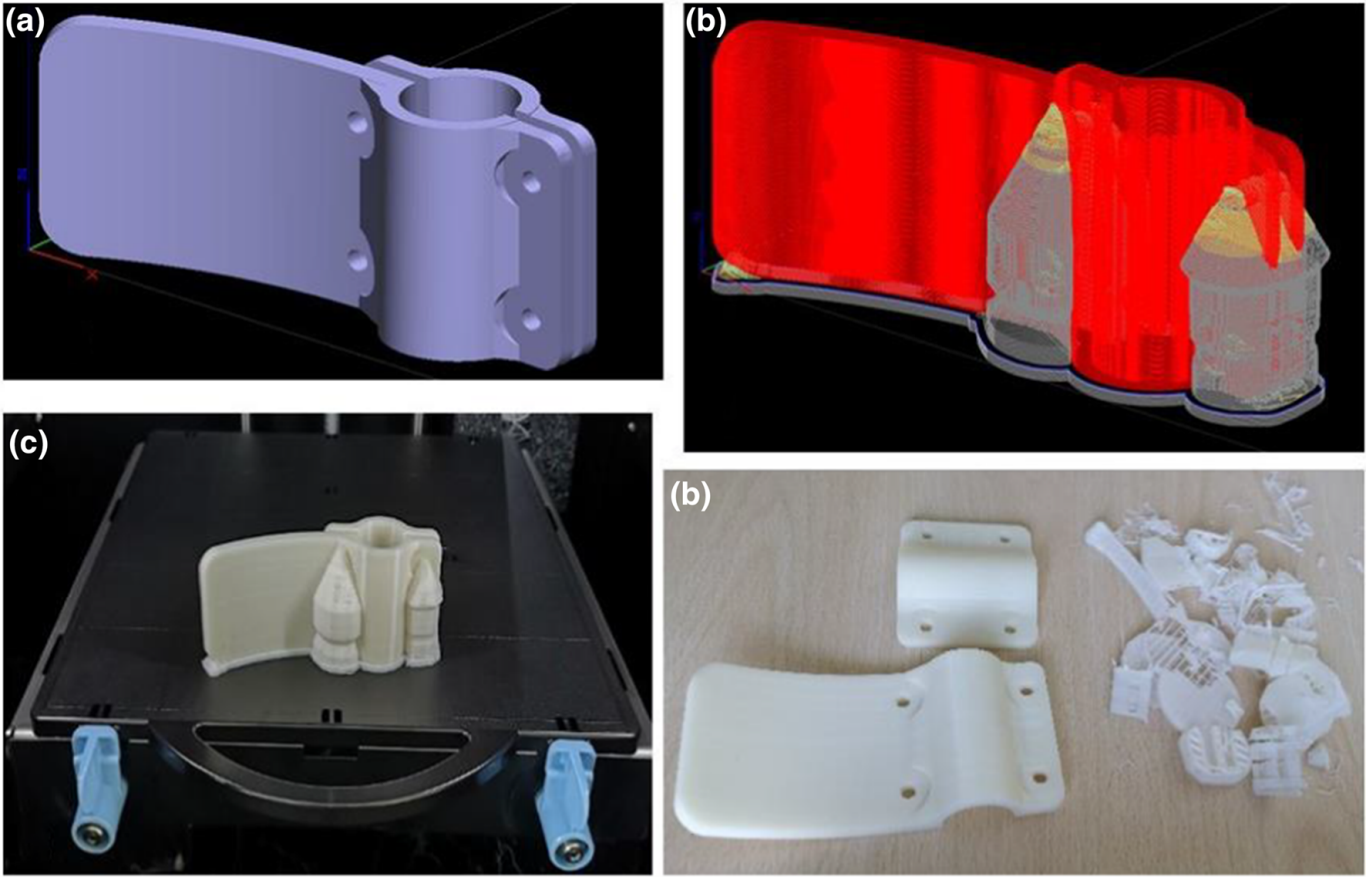
**a** Building orientation plan, **b** Simulating the toolpath and the support material deposition, **c** Getting the part, **d** Removing the support

For what concerns the building orientation, the part has been oriented as in Fig. 9. This orientation optimises the mechanical performance of the part considering the direction of the load, improves the surface roughness, reduces the support material waste, and occupies a smaller area on the printing plate, allowing more parts to be printed in a unique batch.

Therefore, for each type, two physical extensions have been fabricated by varying the filling material options. The Fortus 250 manufactures parts layer by layer through a contour and raster layout and the inner part of each section layer is built through the deposition of parallel fused filaments of ABS [19]. The user can choose among different filling options for obtaining more robust physical parts. In total, four specimens have been printed, two for each type, by varying the filling options among the “Low Density” and the “Solid” options.

## Testing

The four specimens have been printed, installed, and tested. As shown in Table 1 with this kind of geometry around 30% of material and up to 15% of total printing time can be saved by choosing the Low-density option. The total printing time for each part is between 8 and 9 h.

**Table 1:** Time and Material data for printing the specimens

Type	Filling style	Model material (cm^3^)	Support material (cm^3^)	Printing time (h)
Type 1	Low density	28,885	10,491	7:55
Type 1	Solid	40,863	10,491	9:16
Type 2	Low density	30,575	11,381	8:06
Type 2	Solid	43,083	11,379	9:11

Afterwards, the parts have been assembled and tested by potential users to have an idea of how easy is to assemble it and how easy is to open/push/pull doors.No issues have been detected in assembling the system and the result was a very stable connection. This was an expected result since the extension is shaped and dimensioned on the specific handle.

Finally, it must be considered that this kind of product can change radically the way people interact with doors. In the past, some studies have been conducted on the possibility that different handle designs have an impact on the perceived effort in using them. For example, Paschoarelli et al. compared the effort perceived by a sample of 180 potential users in five models of handles [20]. The handle models included in such study had been selected among conventional designs of lever and knob handles, commonly used and already in the background experience of anybody. On the contrary, the extension proposed in this paper is completely new and unknown and it must be tested in terms of usability. Hence, it has to be verified that the resulting product is simple and intuitive to use. In fact, according to the principles for Universal Design presented by Story in [21], easiness and intuitiveness are very important in designing new products.

**Fig. 10:**
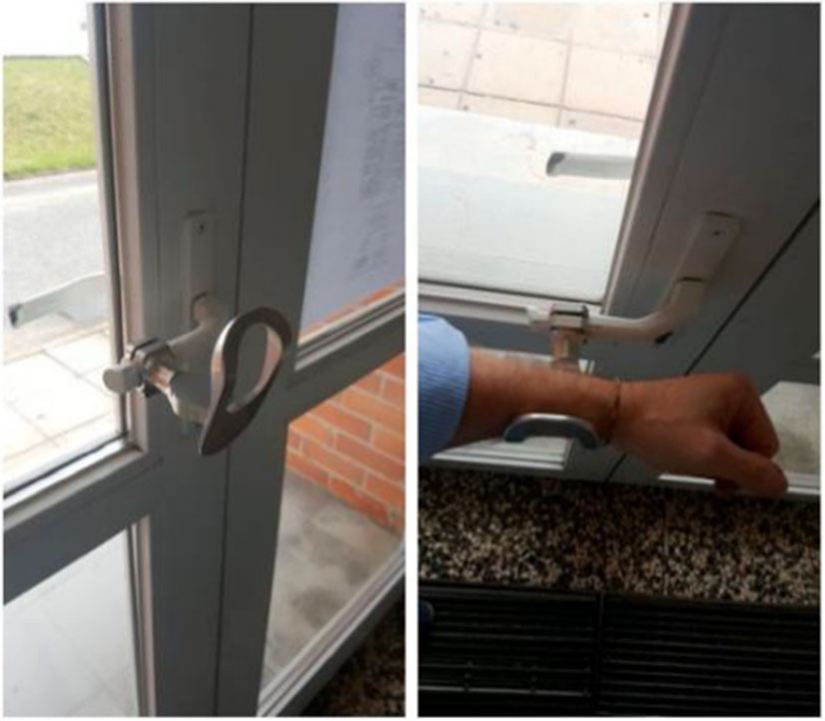
The leaf concept proposed by Maranha et al. [22]

To this aim a leaf-based concept had already been presented and tested by Maranha et al. in [22] revealing that the participants found no problem with the usability of the auxiliary item depicted in Fig. 10.

**Fig. 11:**
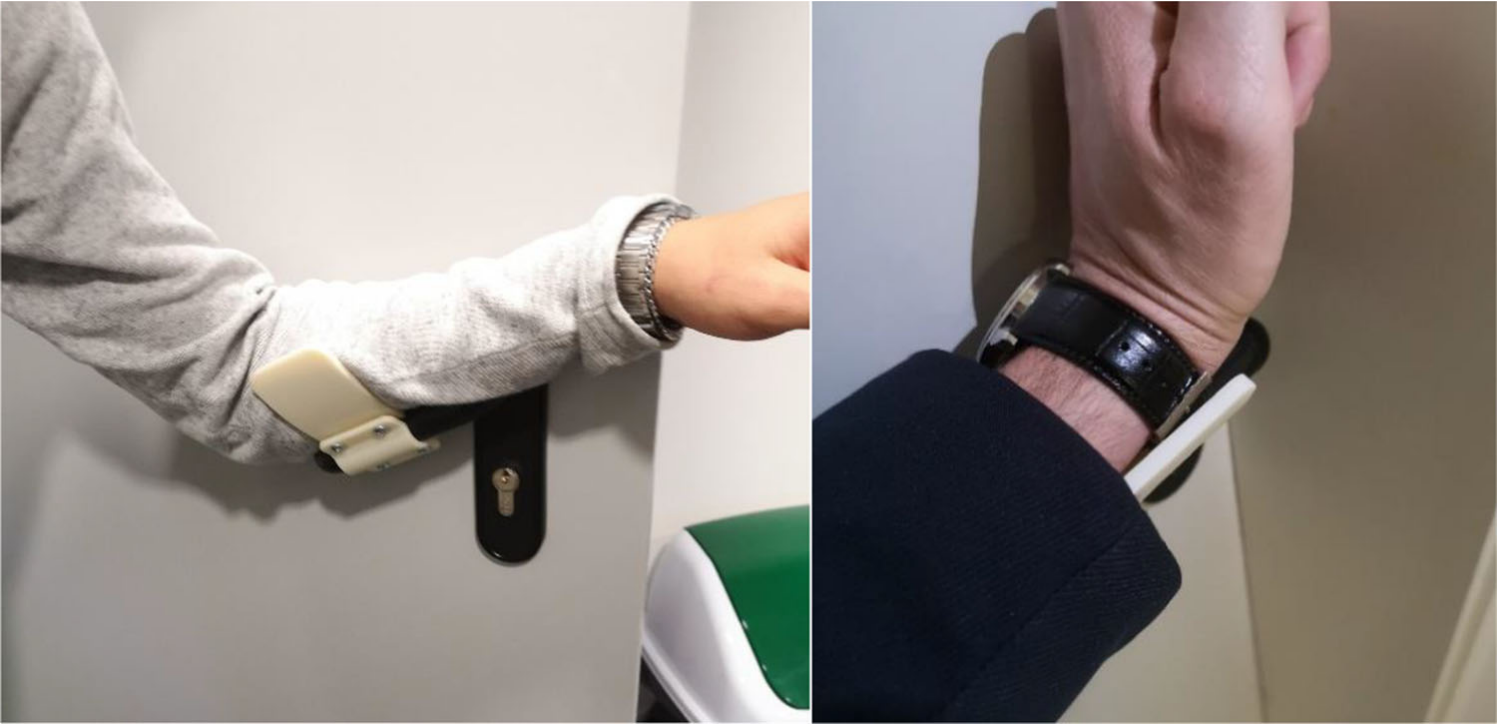
Snapshot of potential user opening the door

In Fig. 11 the device printed in FDM has been installed at the lab door and an opening task is performed by the students. The user/device interface and the user interaction paradigm are the same of the concept proposed in Fig. 10.

## Conclusions

In this paper, a method to semi automatically create 3D printed devices integrated with existing handles that can change the way users interact with doors quickly and cheaply has been proposed. Moreover, it has been demonstrated how a Generative Design approach can help define and structure a modelling algorithm that can generate infinite solutions with an extremely high level of customization. The cost of material and time needed to realise the product have been computed for one of the most common technologies, the FDM. In addition to the reduction of the risk of contagion through surface contact, this solution could also reduce the discomfort of users due to the fear of being infected while touching surfaces in work or public buildings.

Such characteristics make the solution particularly useful, and it could be used even when the emergency will be over reducing the hygienising needs in public buildings and the cost of sanitisation.

Among the limitations of this study, it can be observed that, although the algorithm has demonstrated to work finely even with rough mesh models, the Reverse Engineering process needs to be conducted by operators with some skills in relieving and that a scanner must be owned. Nevertheless, it must also be considered that scanning systems are becoming always easier to use and are starting to be integrated in non-professional devices, such as smart phones and tablets.

In conclusion, three possible future developments of this work have been identified. The first one consists in defining and testing an algorithm for doorknobs or spherical door handles. The second one is the creation of a General User Interface (GUI) for non-CAD experts that need to exploit the model generation features from wherever an internet connection is available. Finally, the shape fitting generation algorithm can be exploited to design handles for people with reduced mobility or for people with disabilities.
